# PD-1 blockade does not improve efficacy of EpCAM-directed CAR T-cell in lung cancer brain metastasis

**DOI:** 10.1007/s00262-024-03837-9

**Published:** 2024-10-03

**Authors:** Jens Blobner, Laura Dengler, Constantin Eberle, Julika J. Herold, Tao Xu, Alexander Beck, Anton Mühlbauer, Katharina J. Müller, Nico Teske, Philipp Karschnia, Dominic van den Heuvel, Ferdinand Schallerer, Hellen Ishikawa-Ankerhold, Niklas Thon, Joerg-Christian Tonn, Marion Subklewe, Sebastian Kobold, Patrick N. Harter, Veit R. Buchholz, Louisa von Baumgarten

**Affiliations:** 1 Department of Neurosurgery, LMU University Hospital, Ludwig Maximilians University (LMU), 81377 Munich, Germany; 2 German Cancer Consortium (DKTK), Partner Site Munich, A Partnership Between the DKFZ Heidelberg and the University Hospital of the LMU, Munich, Germany; 3 Department of Neurology, LMU University Hospital, Ludwig Maximilians University (LMU), 81377 Munich, Germany; 4 Center for Neuropathology and Prion Research, Faculty of Medicine LMU Munich, Ludwig-Maximilians-University (LMU), Munich, Germany; 5 Institute for Medical Microbiology, Immunology and Hygiene, Technical University of Munich, 81675 Munich, Germany; 6 Department of Medicine I, Ludwig-Maximilians-University School of Medicine, Munich, Germany; 7 Department of Medicine III, Ludwig-Maximilians-University School of Medicine, Munich, Germany; 8 Department of Medicine IV, Division of Clinical Pharmacology, LMU University Hospital Munich, Munich, Germany; 9 Bavarian Cancer Research Center (BZKF), 91054 Erlangen, Germany; 10 Division of Neuro-Oncology, Department of Neurosurgery, Ludwig Maximilians University School of Medicine, Marchioninistrasse 15, 81377 Munich, Germany

**Keywords:** CAR T cell, Brain metastasis, Lung cancer, PD-1-blockade

## Abstract

**Background:**

Lung cancer brain metastasis has a devastating prognosis, necessitating innovative treatment strategies. While chimeric antigen receptor (CAR) T-cell show promise in hematologic malignancies, their efficacy in solid tumors, including brain metastasis, is limited by the immunosuppressive tumor environment. The PD-L1/PD-1 pathway inhibits CAR T-cell activity in the tumor microenvironment, presenting a potential target to enhance therapeutic efficacy. This study aims to evaluate the impact of anti-PD-1 antibodies on CAR T-cell in treating lung cancer brain metastasis.

**Methods:**

We utilized a murine immunocompetent, syngeneic orthotopic cerebral metastasis model for repetitive intracerebral two-photon laser scanning microscopy, enabling in vivo characterization of red fluorescent tumor cells and CAR T-cell at a single-cell level over time. Red fluorescent EpCAM-transduced Lewis lung carcinoma cells (^EpCAM/tdt^LL/2 cells) were implanted intracranially. Following the formation of brain metastasis, EpCAM-directed CAR T-cell were injected into adjacent brain tissue, and animals received either anti-PD-1 or an isotype control.

**Results:**

Compared to controls receiving T-cell lacking a CAR, mice receiving EpCAM-directed CAR T-cell showed higher intratumoral CAR T-cell densities in the beginning after intraparenchymal injection. This finding was accompanied with reduced tumor growth and translated into a survival benefit. Additional anti-PD-1 treatment, however, did not affect intratumoral CAR T-cell persistence nor tumor growth and thereby did not provide an additional therapeutic effect.

**Conclusion:**

CAR T-cell therapy for brain malignancies appears promising. However, additional anti-PD-1 treatment did not enhance intratumoral CAR T-cell persistence or effector function, highlighting the need for novel strategies to improve CAR T-cell therapy in solid tumors.

**Supplementary Information:**

The online version contains supplementary material available at 10.1007/s00262-024-03837-9.

## Introduction

Lung cancer remains a fatal disease for the great majority of patients [1]. Approximately, 10% of newly diagnosed patients present with brain metastasis and 25–40% develop brain metastases in the course of disease [2, 3]. Conventional radiotherapy, chemotherapy, and novel treatment strategies including immune checkpoint inhibitors have improved the survival of non-small cell lung cancer (NSCLC) patients in the past decades, but the 5-year survival rate especially of patients with brain metastases remains poor [4].

Adoptive T-cell therapy with genetically modified T-cell that express synthetic receptors on the cell surface to detect and eradicate cancer cells by identifying specific tumor antigens, so-called chimeric antigen receptor (CAR) T-cell, has emerged as one of the most promising cancer immunotherapy modalities[5]. CAR T-cell targeting the B cell lineage antigen CD19 demonstrate remarkable anti-tumor efficacy in the treatment of hematologic cancers. Six CAR T-cell therapies have been granted approval for hematological cancers by the Food and Drug Administration (FDA) since 2017 [6]. Substantial efforts to improve the activity of CAR T-cell against solid tumors including tumors located in the CNS have been made; however, their efficacy remains limited [7–9].

Currently, several clinical trials for CAR T-cell in the treatment of lung cancer are underway [10]. The epithelial cell adhesion molecule (EpCAM) may represent a target for CAR T-cell therapy as EpCAM is overexpressed in about 50% of non-small cell lung cancer and their metastases [11–14]. According to our recent preclinical data, EpCAM-directed CAR T-cell may indeed constitute an effective therapeutic avenue for brain metastases from lung cancer. Additionally, first clinical data show that EpCAM-directed CAR T-cell are both safe and efficacious in the treatment of EpCAM-positive malignancies [15, 16]. However, tumor escape from immune elimination is a hallmark of cancer [17]. Particularly CNS tumors impair CAR T-cell efficacy by an adaptive immune suppressive response including upregulation of immune inhibitory molecules, such as programmed cell death ligand-1 (PD-L1) resulting in immune cell exhaustion [18]. This highlights the need for additional therapeutic strategies to counteract the hostile tumor microenvironment (TME) and overcome tumor heterogeneity.

Immune checkpoint blocking agents (ICB) can elicit durable clinical responses by reactivating an exhausted immune response and are part of standard the standard of care treatment in NSCLC [19]. Therefore, combining CAR T-cell therapy and ICB may produce a synergistic effects: Anti-PD-1 antibody therapy could restore the activity and functional persistence of not only the injected CAR T-cell but also other innate immune cells, including tumor-infiltrating lymphocytes, in the tumor microenvironment and may provide more efficient eradication of cancer cells [20, 21].

In the current study, we combined a chronic cranial window with repetitive two-photon laser scanning microscopy to establish an immunocompetent, syngeneic, orthotopic mouse model of lung cancer brain metastasis. This enables us to the repeatedly visualize fluorescent EpCAM-expressing Lewis lung cancer cells and CAR T-cell at single-cell level. Thereby, we can analyze in vivo dynamics and persistence and were eventually able to evaluate putative synergistic effects of an additional anti-PD-1 treatment.

## Methods

### Cell lines and cell culturing

The LL/2 murine Lewis lung carcinoma cell line was obtained from the European Collection of Authenticated Cell Cultures (ECACC; catalogue number: #90-0201-04). The cells were cultured in DMEM (Sigma-Aldrich; D6429) supplemented with 10% FBS (Sigma-Aldrich; F0804), 100 U/mL penicillin, and 0.1 mg/mL streptomycin (Sigma-Aldrich; P4333) at 37 °C and 5% CO_2_. Regular testing was conducted to detect mycoplasma infection. The cells were maintained in culture for up to four weeks after thawing to minimize genetic drift. No evidence of mycoplasma, squirrel monkey retrovirus, or interspecies contamination was found.

### Tumor cell line generation

The generation of red fluorescent LL2/EpCAM cells was carried out as previously described [16]. In brief, the lentiviral expression vector pLVX-IRES-neoR (LentiX-Bicistronic Expression System; catalog number #632,181; TaKaRa Clontech) was utilized to clone a PCR product containing the coding sequence of tdTomato (vector tdTomato; catalog number #63–2531; TaKaRa Clontech). The resulting construct pLVX-tdTomato-IRES-Neo, incorporating a resistance sequence for G418-sulfate, was validated through restriction enzyme digestion and Sanger sequencing [22]. Subsequently, LL/2 cells were transfected with pLVX-tdTomato-IRES-Neo using lipofection (Lipofectamine 3000; Thermo Fisher Scientific). Enrichment was achieved through cultivation in selection medium containing G418-sulfate and Geneticin (#A2912; Biochrom), along with repetitive FACS sorting. As previously described in detail [23], ^tdt^LL/2 cells were stably transduced with a pMXs vector containing the full-length murine EpCAM (UNIPROT entry: #Q99JW5) cDNA, resulting in the creation of the EpCAM-overexpressing cell line ^EpCAM/tdt^LL/2. EpCAM expression by ^EpCAM/tdT^LL/2 tumor cells was confirmed using immunofluorescence staining.

### Retroviral *CAR* vector

In our study, the anti-EpCAM CAR construct was used as previously described [24]. The murine EpCAM antigen (clone G8.8) is recognized by a single-chain variable fragment fused to the transmembrane and signaling domains of murine CD28 and murine CD3ζ in a pMP71 backbone. The anti-EpCAM-CAR-GFP construct comprises the anti-EpCAM-CAR fused to GFP through a self-cleaving 2A sequence. Transduction was conducted to generate GFP-fluorescent, EpCAM-directed CAR T-cell (^GFP/EpCAM^CAR T-cell). For the generation of ^GFP^T-cell, a pMP71 retroviral vector containing only eGFP was utilized.

### T-cell isolation and transduction

CAR T-cell were generated as described previously [16]. Briefly, spleens of naïve C57BL6/j mice were harvested directly after cervical dislocation and a single-cell suspension was obtained using a 35 µm cell strainer (Greiner Bio-One; 542,070). Erythrocytes underwent lysis utilizing ACK lysis buffer (comprising 150 mM NH4Cl, 10 mM KHCO3, and 100 μM Na2EDTA) for a duration of 2 min. Following this, cells were rinsed with PBS. The transduction of primary murine T-cell was carried out in accordance with prior descriptions [24]. In brief, splenocytes were cultured in RPMI 1640 containing 10% fetal calf serum (FCS), 0.025% l-glutamine, 0.1% HEPES, 0.001% gentamicin, and 0.002% streptomycin supplemented with 25 U/mL IL-2 and stimulated overnight with anti-mouse CD3 antibodies (1:1,000, clone 145–2C11; BD Pharmingen) and anti-mouse CD28 antibodies (1:5,000, clone 37.51; BD Pharmingen). Supernatants containing the corresponding virus were used for the transduction procedure. During virus generation, T-cell were stimulated with Dynabeads Mouse T-Activator CD3/CD28 (#11-452D, Thermo Fisher). Transduced murine T-cell were cultured in murine T-cell medium enriched with recombinant human IL-7 (#200–007; Peprotech), IL-15 (#200–015; Peprotech), and β-mercaptoethanol (M6250-100; Sigma-Aldrich) for a maximum period of 7 days. Following retroviral transduction and expansion, ^EpCAM/GFP^CAR T-cell and ^GFP^T-cell were selected by CD3- and GFP-positivity utilizing a MoFlow Cell Sorter (Beckman Coulter) after exclusion of dead cells.

### Animals

Male C57BL/6 J wild-type mice were purchased from Charles River (Sulzfeld, Germany) at the age of 8–12 weeks and were kept under pathogen-controlled conditions and were used for further experiments. All animal experiments were approved by the local governmental animal care committee (permission number: 02–20-44) of the Ludwig-Maximilians-University Munich, Germany. The experiments were conducted in compliance with European legislation regarding animal protection and adhered to NIH Guidelines (NIH Publication #85–23 Rev. 1985).

### Surgical Procedure and postoperative care

Preparation of a chronic cranial window was performed as previously described [22, 25, 26]. Briefly, a circular section of the skull (with a 5.5 mm diameter) was excised using a sterile carbon steel microdrill. Following this, the dura mater was separated from the below leptomeninges using two forceps in order to prevent dural fibrosis and optimize image resolution. Subsequently, the brain surface was coated with PBS, and a sterile round cover-glass was applied. For enhanced head positioning during imaging, a custom-made ring (made of polyether ether ketone [PEEK]) was securely affixed to the cranial bone using acrylic dental glue (Cyano Veneer). Buprenorphine (0.1 mg/kg; q8h) was administered for two consecutive days to ensure postoperative analgesia. To minimize postoperative microglia activation and preserve the integrity of intracranial microcirculation, a recovery period of 21 days was observed before proceeding with additional experiments.

### Intraparenchymal tumor cell inoculation and *CAR* T-cell injection

After being resuspended in 1 µL PBS, stereotactically injection of 2.5 × 10^3 EpCAM/tdt^LL/2 was performed into the left hemisphere at predefined coordinates (1 mm lateral to the sagittal sinus and 2 mm posterior to the bregma; intraparenchymal depth: 1.3 mm). Depending on the experimental setup, injection was done either after borehole placement (survival analysis) or careful removal of the chronic cranial window (in vivo imaging analysis).

At day 4 after tumor cell inoculation, mice were randomized to the different treatment groups. For immune checkpoint blockade, 250 µg anti-PD-1 (RMP1-14, BioXCell) per mouse or equivalent doses of isotype control antibodies (2A3, BioXCell) were administered by intraperitoneal (i.p.) injection in 200 µl PBS every 3 days starting 4 days after tumor cell injection. The crossing of the blood–brain barrier and the intracerebral efficacy of the aPD-1 in vivo antibodies was shown recently in a murine brain tumor model [27]. Seven days after tumor cell injection, 2 × 10^5 EpCAM/GFP^CAR T-cell or ^GFP^T-cell resuspended in 1 µL PBS were injected 1 mm posterior to the injection point (intraparenchymal depth: 1.3 mm) either after borehole placement (survival analysis) or careful removal of the chronic cranial window (in vivo imaging analysis).

### Two-photon laser scanning microscopy (TPLSM)

TPLSM data were acquired using a Multiphoton TrimScope II system (LaVision BioTec) coupled with an upright Olympus microscope featuring a TiSA Coherent Chameleon Ultra-II-Femtolaser (wavelength 800 to 1,080 nm; Spectra Physics, Newport). The data were obtained using either a 4 × objective (NA 0.28; Olympus XLFluor 4 × /340) or a 20 × water immersion objective (numerical aperture [NA] 0.95; Olympus XLUMPlanFl). During the imaging process, the mice were positioned on a heating mat and anesthetized with isoflurane in oxygen. The concentration of isoflurane was adjusted to 1.0 to 2.0% based on the breathing rate.

To minimize movement during imaging, a PEEK ring was glued adjacent to the chronic cranial window and fixed using a custom-made fixation device. For enhanced visualization of intra- and extratumoral cerebral vessels, 0.1 ml of fluorescein isothiocyanate (FITC)-dextran (2 MDa molecular mass) was intravenously injected at a concentration of 10 mg/ml. Imaging starts at the surface (determined by detecting arachnoid fibers using second harmonic imaging) and progressed every 5 µm up to a depth of 400 µm. Image resolution was set at 1024 × 1024 pixels with a wavelength of 920 nm.

Three-dimensional (3D) stacks and dynamic images were captured using x/y/z-dimensions of 450 × 450 × 400 μm. Dynamic analyses involved 3D image stacks with x/y/z-dimensions of 450 × 450 × 66 μm. Images were acquired repetitively over 20 min, starting 100 µm below the cortical surface to ensure intratumoral imaging.

### Image analysis

ImageJ/Fiji and Imaris (Bitplane AG) were used for image processing and analysis. To ensure an unbiased processing approach, raters were kept blinded to group allocation until the final data analysis. To measure tumor size using in vivo microscopy, we quantified the number of fluorescent pixels of the 2D tumor area using epifluorescence microscopy. CAR T-cell were identified by their green fluorescent signal, while tumor cells were recognized by their red fluorescent signal.

### Immunofluorescence and multiplex analysis

Following cardiac perfusion, the brains were removed and fixed in 4% PFA. To facilitate dehydration, the samples underwent a series of sucrose incubations until equilibrium was reached. Subsequently, the brains were exposed to the gas phase of liquid nitrogen for 5 min before being stored at -80 °C. Finally, the brains were sectioned into 15-μm-thick slices with a spacing of 495 µm.

To quantify (CAR) T-cell counts, sections were stained with a chicken anti-GFP antibody (#AB13970; 1:200, Abcam) to specifically detect the GFP signal associated with (CAR) T-cell. The secondary antibody used was a goat anti-chicken AlexaFluor® 488 antibody (#AB150169; 1:200, Abcam). For investigations related to the immunosuppressive tumor microenvironment, sections underwent additional staining with rat anti-CD3 antibody (MAB4841, 15 µg/mL R&D). The applied secondary antibodies were chicken anti-rabbit AlexaFluor® 647 antibody (#A21443; 1:100, Invitrogen) and chicken anti-rat AlexaFluor® 647 antibody (#A21472; 1:100, Invitrogen), respectively. Following an overnight incubation in a humidified box at 4 °C with the primary antibodies, secondary antibody labeling occurred at room temperature for 1 h. The staining of cell nuclei was achieved using DAPI (#236,276; 1:1000, Roche).

The sections were analyzed using a Zeiss AxioImager M2 upright microscope from Carl Zeiss Microscopy. To calculate the tumor volume and CAR T-cell density through immunofluorescence, the tumor was manually outlined based on the fluorescence signal using the Zen Lite software package (version 2.3; Carl Zeiss Microscopy). The total tumor area per slice was then multiplied by a thickness of 495 μm, and the sum of these values yielded the overall tumor volume. The CAR T-cell density was calculated by dividing the total number of cells by the total tumor volume. Additionally, intratumoral CAR T-cell were categorized based on their location, either in the tumor core or the border zone (0–10 µm from the outer tumor edge). To analyze CD3 and Iba1, one to two random tumor slices per mouse were selected, and the density of CD3-positive cells was measured per tumor area. Detected cells were classified based on their location in either the tumor core or border zone.

For multiplexed immunofluorescence imaging, FFPE tissue sections were deparaffinized and rehydrated. Antigen retrieval was conducted utilizing a pressure cooker and a 1 × citrate buffer solution at pH 6.0. Following this step, sections underwent staining using the Opal Polaris 7 Color Manual IHC Detection Kit from Akoya Biosciences. Primary antibodies were applied at a dilution of 1:100: TIM-3 (Cell Signaling, 83,882), PD-L1 (Cell Signaling, 13,684), PD-1 (Cell Signaling, 84,651), LAG3 (Proteintech, 16,616–1-AP), EpCAM (Thermo Fisher, 14–9326-82). Full fluorescent slide scans were taken via the PhenoImager Fusion (Akoya Biosciences). Image analysis was carried out by using inForm analysis software (Akoya Biosciences) for spectral unmixing alongside the open-source software QuPath for image analysis. Regions of interest (ROIs) were delineated using polygonal shapes. Manual exclusion of tissue folds and staining artifacts was performed. Cells were segmented based on the DAPI signal utilizing the Cell detection tool. Further, cells were phenotyped by establishing single measurement classifiers, which were manually configured and optimized for each stain.

### Flow cytometry and fluorescence-activated cell sorting (FACS)

Flow cytometry and cell sorting were performed on a Beckman Coulter MoFlo Astrios cell sorter. To validate homogeneous expression of tdTomato, LL/2 tumor cells underwent flow cytometry analysis. During CAR T-cell production, ^EpCAM/GFP^CAR T-cell and ^GFP^T-cell were selected based on CD3- and GFP-positivity using a MoFlow Cell Sorter (Beckman Coulter) after retroviral transduction and expansion. Dead cells were excluded.

### Study protocol

**Fig. 1:**
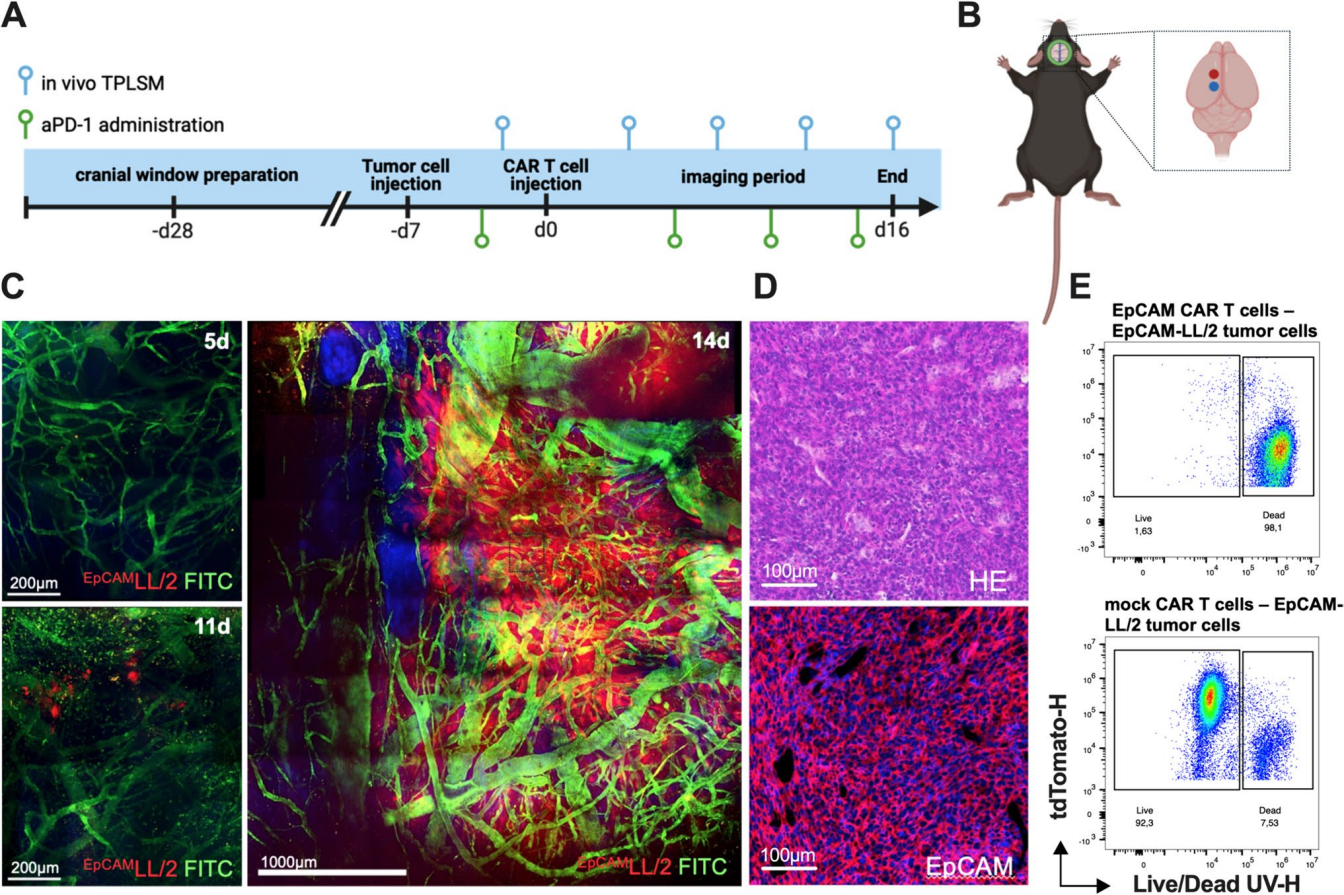
Experimental protocol. **a** Schematic illustrating the experimental setup, including chronology of tumor cell injection, CAR T-cell injection, ICB therapy, and imaging methodologies. **b** Illustration of the injection sites of tumor cells and (CAR) T-cell, respectively. **c** Intracerebral tumor growth following stereotactic implantation of ^EpCAM/tdT^LL/2 tumor cells (red). Blood vessels are highlighted after i.v. injection of FITC-dextran (green). Images represent mosaics of multiple maximum intensity projections with 400 µm depth from the brain surface (Scale bar 200 µm left pictures, 1000 µm right picture). Note that the day count refers to the day after tumor cell injection. **d** Hematoxylin and eosin staining as well as immunofluorescence staining of EpCAM on brain tumor tissue. Scale bar 100 µm. **e** Flow cytometry of ^EpCAM/tdT^LL/2 tumor cells after 48 h of co-culturing with ^EpCAM/GFP^CAR T-cell and ^GFP^T-cell, respectively

After the preparation of the cranial window, the mice were allowed to recover for a period of at least 21 days. To monitor tumor development using TPLSM, 2500 tumor cells were injected at standardized coordinates into the brain parenchyma. On day 4 after tumor cell inoculation, baseline epifluorescence imaging is conducted, and mice are then randomly assigned to one of the four groups: one receiving ^EpCAM/GFP^CAR T-cell and anti-PD-1 (*n* = 8), one receiving ^GFP^T-cell and anti-PD-1(*n* = 9), one receiving ^EpCAM/GFP^CAR T-cell and IgG (*n* = 8), and one receiving ^GFP^T-cell and IgG (*n* = 8). CAR T-cell injection was performed seven days after tumor cell injection. Intraperitoneal injection of anti-PD-1 and IgG, respectively, started 3 days prior to CAR T-cell administration and continued every 3 days until termination of the experiment (Fig. 1a). In vivo microscopy was performed on day 4, 7, 10, 13, and 16 after CAR T-cell injection. On day -2, 2D-epifluorescence imaging was done to confirm tumor take and for baseline tumor measurement. Animals were killed by intracardiac injection of 0.9% NaCl solution followed by PFA 4% at the end of in vivo microscopy (day 16 after CAR T-cell injection) or when termination criteria were met. Brains were excised for immunofluorescence experiments. Burr hole injection of tumor cells and CAR T-cell, respectively, was done for survival experiments. On this occasion, animals were killed if termination criteria were met.

### Statistics

Statistical analysis was performed using GraphPad Prism software (v9.0). Unless stated otherwise, normal distribution was assessed using the D’Agostino–Pearson test. Differences were evaluated using the Student’s t test for parametric data or the Mann–Whitney U test for nonparametric data. Associations among categorical variables were assessed using the χ2 test. All data are presented as mean ± SEM. Absolute numbers and percentages are used to describe categorical variables. The Kaplan–Meier method was used for survival analysis, accompanied by the log-rank test. The significance threshold was set at *p* ≤ 0.05. The main manuscript contains all the necessary data to validate the findings of this study.

## Results

### Development of a robust murine model for repetitive imaging of lung *cancer* brain metastases

Microsurgical implantation of a chronic cranial window was well tolerated, enabling repetitive in vivo imaging at identical coordinates through two-photon laser scanning microscopy. After inoculation of 2.5 × 10^3 EpCAM/tdt^LL/2 tumor cells, all mice (*n* = 8 per group) had visible tumors at day 5 after tumor cell injection (Fig. 1a, b). These cells form solitary lesions with a subsequent exponential growth pattern (Fig. 1c). The window quality and fluorescence of tumor cells and CAR T-cell was persistent, facilitating the visualization of the stereotactically implanted tumor cells as well as CAR T-cell at a single-cell resolution over several weeks. EpCAM expression by tumor cells was confirmed using immunofluorescence staining (Fig. 1d).

### In vitro* cytotoxicity of EpCAM-directed CAR T-cell.*

To ensure optimal in vivo functionality, physiological cytokine and chemokine production, proliferation, and migration patterns, we generated murine GFP-expressing ^EpCAM/GFP^CAR T-cell using murine transmembrane and costimulatory domains. To evaluate the specific cytotoxicity of this newly generated EpCAM CAR, we co-cultured FACS-sorted EpCAM-directed CAR T-cell with ^EpCAM/tdt^LL/2 target cells. To control unspecific T-cell receptor (TCR)-mediated xenogeneic cytotoxicity of murine T-cell independent of CAR-mediated effects, we conducted a comparison of the killing activities of T-cell that were transduced with the same vector but lacked the CAR construct. After a 48-h co-culturing period, only EpCAM-directed CAR T-cell show dose-depending cytotoxicity that was not detectable for sham-transduced T-cell illustrating the ability of EpCAM-directed CAR T-cell to specifically target and kill ^EpCAM/tdt^LL/2 target cells without any bystander cells (Fig. 1e).

### In vivo* behavior of CAR T-cell after intraparenchymal injection*

**Fig. 2:**
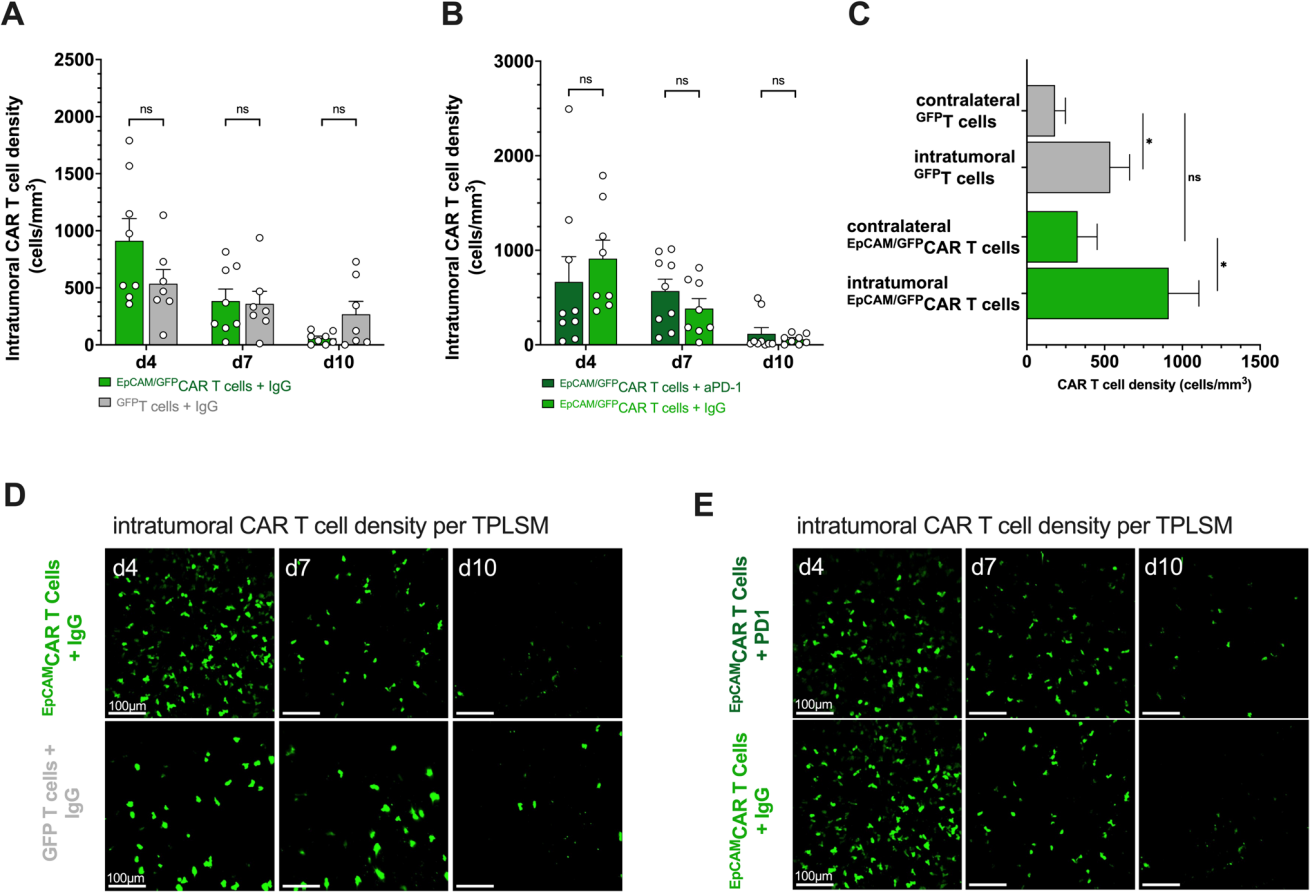
TPLSM of ^EpCAM/GFP^CAR T-cell and ^GFP^T-cell after intraparenchymal injection 7 days after tumor cell inoculation. **a**, **b** Intratumoral (CAR) T-cell density (cells/mm^3^) on d4, d7, and d10 after local injection of ^EpCAM/GFP^CAR T-cell (+ IgG isotype (light green; *n* = 8) or anti-PD-1 antibodies (dark green, *n* = 8)) and ^GFP^T-cell (+ IgG isotype (*n* = 7)), respectively, as determined by two-photon laser scanning microscopy. **c** Comparison of (CAR) T-cell densities (cells/mm^3^) on day 4 after injection within the tumor and the contralateral, non-tumor-bearing hemisphere as assessed by TPLSM in maximum intensity projection (MIP). All results are displayed as mean ± SEM. **d**, **e** Representative images of EpCAM-directed CAR T- (*n* = 8) and undirected T-cell (*n* = 7) with isotype control IgG (D) or in combination with intraperitoneal anti-PD-1-treatment **e** on d4, d7, and d10 after intraparenchymal administration using in vivo TPLSM in MIP with 400 µm depth from the brain surface. Scale bars 100 µm. Mean ± SEM

As previously demonstrated by our group, intracranial but not intravenous injection of ^EpCAM/GFP^CAR T-cell results in sufficient tumor control [16]. Therefore, we sought to analyze intracerebral CAR T-cell trafficking and anti-tumor efficacy after intraparenchymal injection. Seven days after tumor cell inoculation 2 × 10^5 EpCAM/GFP^CAR T-cell were stereotactically administered into the brain parenchyma 1 mm adjacent to the tumor. To demonstrate CAR specificity, control mice were injected with ^GFP^T-cell of similar numbers. Anti-PD-1 treatment was started 3 days before CAR T-cell injection to provide sufficient systemic drug levels. Quantifying intratumoral CAR T-cell density per TPLSM showed that both ^EpCAM/GFP^CAR T-cell and ^GFP^T-cell accumulate intratumorally over time on day 4 following injection. Intratumoral numbers of ^EpCAM/GFP^CAR T-cell exceed those of ^GFP^T-cell illustrating target tropism and successful tumor infiltration (Fig. 2a). Although a relevant number of ^EPCAM/GFP^CAR T-cell were also found in the contralateral hemisphere, no significant differences in (CAR) T-cell densities were found compared to ^GFP^T-cell-treated controls (Fig. 2c). This might indicate enhanced proliferation or migration of intratumoral ^EPCAM/GFP^CAR T-cell rather than passive diffusion from the injection site alone.

### Effects of immune checkpoint blockade on the efficacy of *CAR* T-cell therapy

**Fig. 3:**
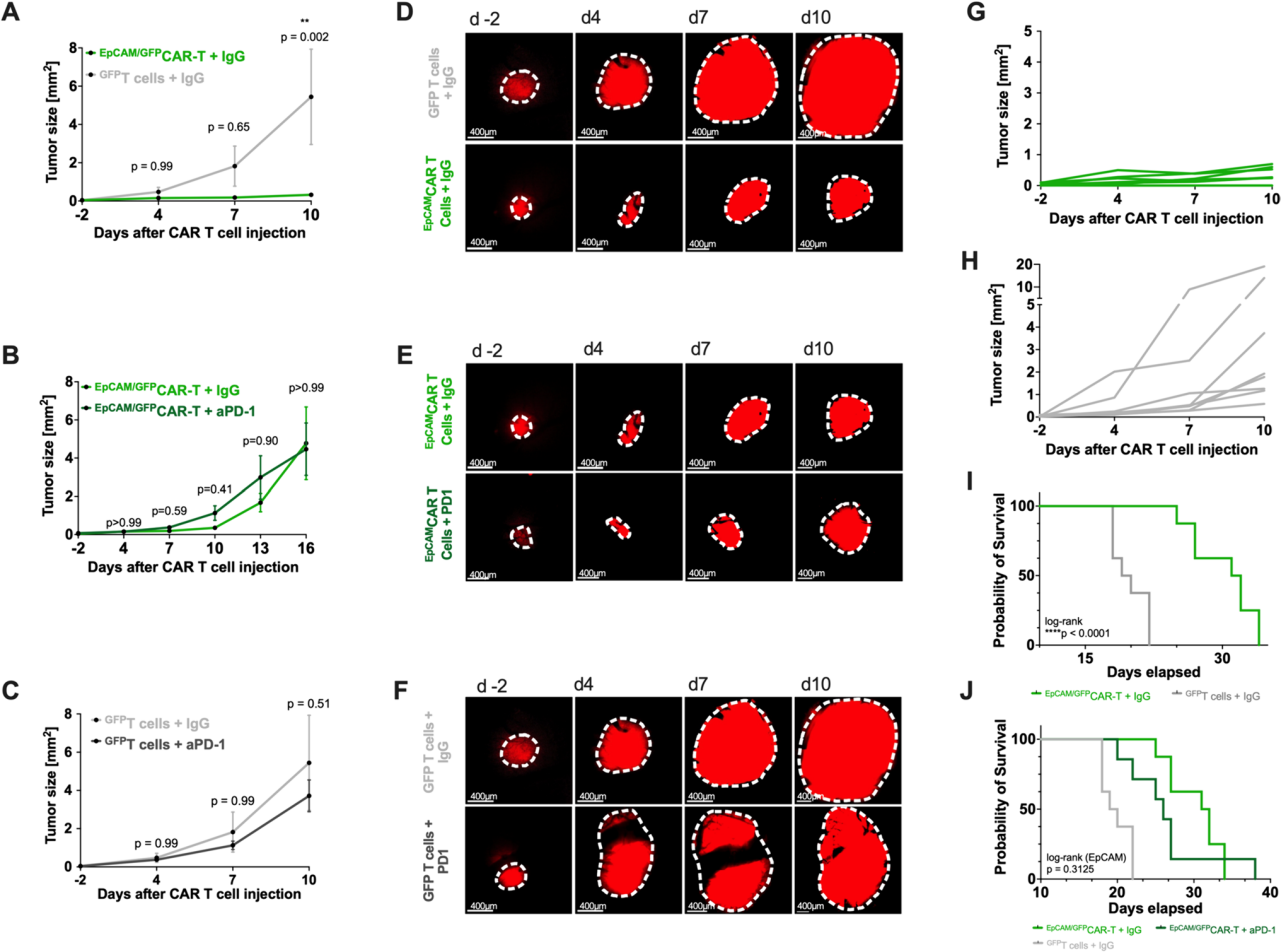
Tumor growth and survival after intraparenchymal injection of (CAR) T-cell with and without concomitant aPD-1 treatment. **a**, **b**, **c** Summarized tumor areas (mm^2^) of *n* = 8 animals receiving locally injected ^EpCAM/GFP^CAR T-cell + IgG (A, B, light green) / + aPD-1 (B, dark green) and *n* = 8 animals receiving ^GFP^T-cell + IgG (A, C, light gray) / + aPD-1 (C, dark gray), respectively. Growth behavior was determined by in vivo microscopy using epifluorescence. Mean ± SEM. ***p ≤ 0.0005. **d**, **e**, **f** Brain tumor growth illustrated by one representative animal on day -2, 4, 7, and 10 after local (CAR) T-cell administration and intraperitoneal anti-PD-1/IgG isotype injection measured by TPLSM using epifluorescence. Tumor cells are visualized by their red fluorescent signal. Scale bars 400 µm. **g**, **h** Individual tumor areas (mm^2^) of *n* = 8 ^EpCAM/GFP^CAR T-cell + IgG (G) and *n* = 8 ^GFP^T-cell + IgG (H), respectively, measured by in vivo TPLSM using epifluorescence on days − 2, 4, 7 and 10 after local injection. **i**, **j** Kaplan–Meier survival estimates for tumor-bearing mice (injected seven days prior to local (CAR) T-cell administration) treated with either ^EpCAM/GFP^CAR T-cell + aPD-1 (dark green; *n* = 8), ^EpCAM/GFP^CAR T-cell + IgG isotype antibody (light green; *n* = 8) or ^GFP^T-cell + IgG isotype antibody (light gray, *n* = 8). Log-rank test, ****p < 0.0001

Particularly in solid tumors, adoptively transferred T-cell face an immunosuppressive microenvironment leading to T-cell exhaustion. Therefore, we aimed to elucidate whether repetitive intraperitoneal administration of PD-1-blocking antibodies may restore T-cell effector function, reduce tumor growth, and prolong survival. Tumors in mice treated with ^GFP^T-cell exhibited an exponential growth pattern, resulting in substantial tumor sizes by day 10 after CAR T-cell injection, with 87.5% (7/8) of the animals displaying tumor sizes exceeding 1 mm^2^ (Fig. 3h). In contrast, mice treated with ^EpCAM/GFP^CAR T-cell demonstrated a reduced growth rate, and none of the animals (0/8) reached a tumor size greater than 1 mm^2^ (Fig. 3g). By day 10 following CAR T-cell injection, there was a reduction of tumor growth (0.32 mm^2^ ± 0.26 vs. 5.44 mm^2^ ± 7.04; *p* = 0.002) (Fig. 3a, d, g). This reduction was accompanied by the intratumoral accumulation of CAR T-cell (Fig. 2a, d). In one out of eight animals (12.5%), the injection of ^EpCAM/GFP^CAR T-cell resulted in a complete regression of the tumor and no visible tumor was observed in the in vivo imaging from day 4 until the end of the experiment (Fig. 3g). Interestingly, additional anti-PD-1 treatment did not increase intratumoral ^EpCAM/GFP^CAR T-cell density or anti-tumor efficacy, resulting in a similar growth pattern compared to animals receiving the IgG isotype control antibody (Figs. 2b, e and 3b, c, e, f). In a separate set of experiments focused on overall survival, burr hole trepanation was performed instead of cranial window implantation. As previously demonstrated, we observed a reduction in tumor growth after intraparenchymal injection of ^EpCAM/GFP^CAR T-cell, which was accompanied by a survival benefit compared to ^GFP^T-cell (Fig. 3i). It is noteworthy that concomitant anti-PD-1 treatment was not able to ameliorate tumor-induced T-cell exhaustion, resulting in similar growth patterns and overall survival rates between the ^EpCAM/GFP^CAR T-cell/anti-PD-1 and ^EpCAM/GFP^CAR T-cell/IgG-treated animals, respectively (Fig. 3b, e and j).

### In vivo* CAR T-cell dynamics and spatial distribution below visualizable depths*

**Fig. 4:**
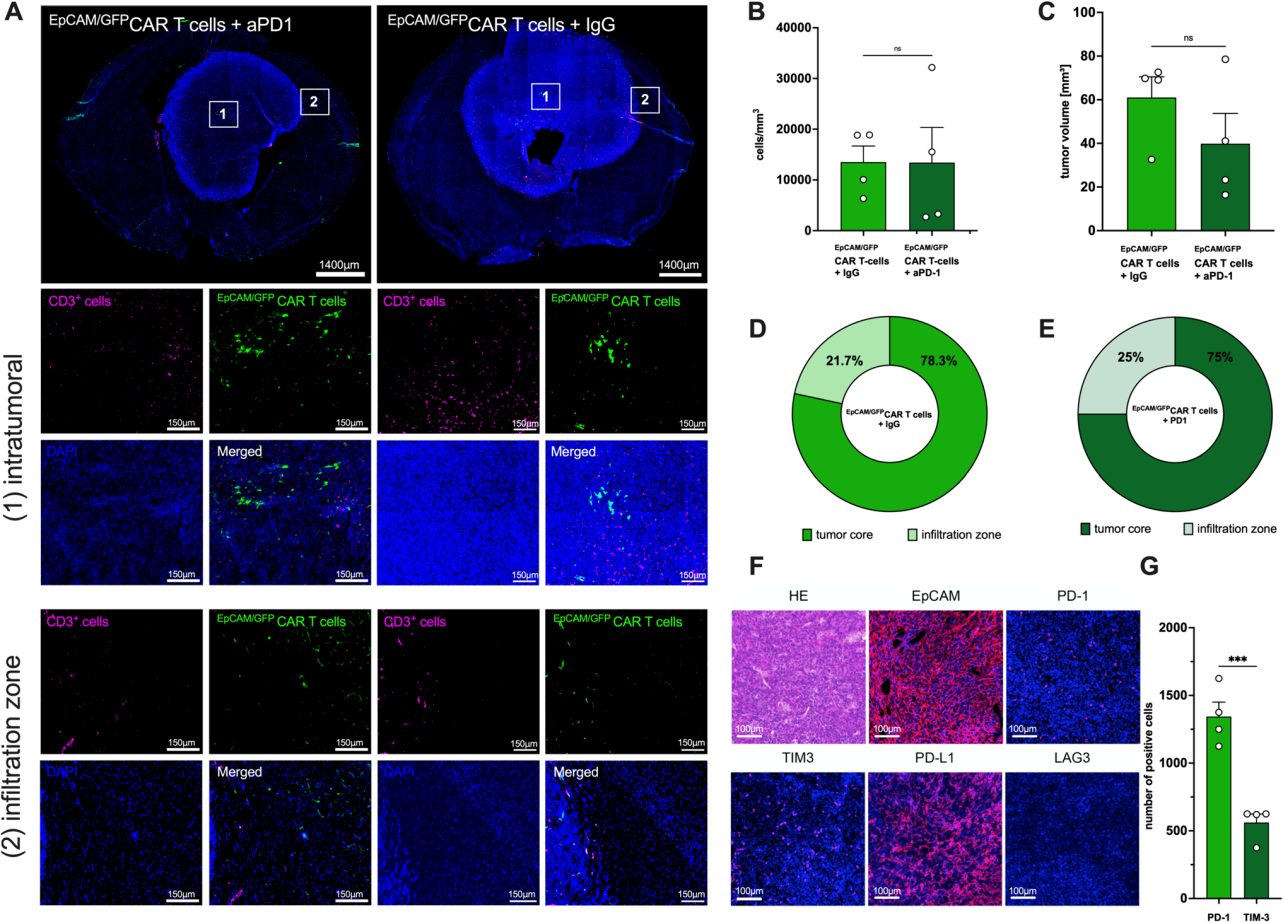
Characterization of intratumoral CAR T-cell dynamics below visible depths using immunofluorescence. After termination of the experiment brains get excised and stained for (CAR) T-cell density. **a** Histological sections of brains after tumor cell and ^EpCAM/GFP^CAR T-cell injection and intraperitoneal aPD-1 and IgG administration, respectively. Sections were stained with an antibody against GFP to identify CAR T-cell (green), against CD3 to visualize T-cell (pink) and DAPI for cell nuclei (blue). CAR T-cell density within the tumor and at the infiltration zone was analyzed. Please note: Tumor size does not differ significantly between both groups. Scale bars 1400 µm (top images) and 150 µm (small images). **b** Intratumoral CAR T-cell density (cells/mm^3^) at the end of the experiment with (*n* = 4) and without (*n* = 4) concomitant aPD-1 treatment. **c** Tumor volume (mm^3^) of ^EpCAM/GFP^CAR T-cell treated animals with and without concomitant anti-PD-1 treatment. Mean ± SEM. **d**, **e** Percentage distribution of CAR T-cell within the tumor and at the infiltration zone determined by immunofluorescence. **f** Immunofluorescence staining of tumor sections. EpCAM expression by tumor cells was confirmed. Expression levels of the immune checkpoint molecules PD-1, PD-L1, TIM-3, and LAG-3 are illustrated in pink. Scale bars 100 µm. g Quantification of PD-1- and TIM-3-positive cells. ***p < 0.001

Repeated in vivo two-photon laser scanning microscopy provides reliable imaging of tumor-immune cell interactions down to a depth of 400 µm. To validate our 2-photon imaging findings and gain further insights into the anti-tumor effects and spatiotemporal distribution of locally injected CAR T-cell, we conducted immunofluorescence analyses of excised brains from animals treated with CAR T-cell, with and without simultaneous anti-PD-1 treatment. The brains of mice treated with ^EpCAM/GFP^CAR T-cell (± anti-PD-1/IgG antibodies) and mice-treated ^GFP^T-cell (± anti-PD-1/IgG antibodies) were collected between day 10 and day 16 following intracerebral CAR T-cell injection when mice met termination criteria. Tumors were found in 15 of 16 (93.8%) mice treated with ^EpCAM/GFP^CAR T-cell (± anti-PD-1/IgG antibodies) and in all mice of ^GFP^T-cell (± anti-PD-1/IgG antibodies). Immunofluorescence analysis of tumor volumes did not reveal differences between mice treated with ^EpCAM/GFP^CAR T-cell and concurrent aPD-1 treatment and those given ^EpCAM/GFP^CAR T-cell with the isotype control antibody (40 mm^3^ ± 27.8 mm^3^ vs. 61 mm^3^ ± 19 mm^3^, *p* = 0.25) (Fig. 4c) further confirming our results of in vivo microscopy. Strong EpCAM expression was observed in LL/2 brain tumors until the end of the experiment, making it unlikely that antigen loss serves as a mechanism of therapy resistance (Fig. 4f). Furthermore, we compared intratumoral density of ^EpCAM/GFP^CAR T-cell in animals with and without concurrent aPD-1 treatment. Consistent with our prior in vivo microscopy results, additional immune checkpoint blockade demonstrated no relevant impact on intratumoral infiltration by CAR T-cell (Fig. 4a, b). Expression of both PD-1 and PD-L1 was observed in animals treated with either ^EpCAM/GFP^CAR T-cell or ^GFP^T-cell. This suggests that the absence of these molecules is not responsible for the lack of a synergistic effect with anti-PD-1 treatment (Fig. 4f). To determine whether other immune checkpoints might contribute to (CAR) T-cell exhaustion, we examined TIM-3 and LAG-3 expression. While LAG-3 was not present in our model, TIM-3 expression was observed on the cell surface, albeit at lower levels compared to PD-1 (Fig. 4g). Nonetheless, (CAR) T-cell silencing via this pathway may be a potential mechanism.

Next, we analyzed the spatial distribution of ^EpCAM/GFP^CAR T-cell (± anti-PD-1/IgG antibodies) within the tumor between both experimental groups. Even though there was no difference between both groups, elevated quantities of CAR T-cell were detected within the tumor core in contrast to the tumor border (Fig. 4d, e). However, it should be noted that the absolute number of CAR T-cell at the end of the experiment was limited.

## Discussion

Lung cancer is the leading cause of cancer deaths, with approximately 20% of patients diagnosed with metastatic disease [28]. Although therapeutic strategies for lung cancer brain metastases have advanced, CNS spread still significantly affects survival and quality of life [29]. While immune checkpoint inhibitors show promise in some cases, most patients do not respond to these therapies, emphasizing the need for new treatments [30]. Challenges in cellular-based approaches for solid brain tumors include the consistent identification of targets [31].

We used a fully immunocompetent murine model of lung cancer brain metastases and combined a chronic cranial window with repeated in vivo TPLSM to explore real-time dynamics of CAR T-cell at a single-cell level during combined administration of anti-PD-1 and CAR T-cell. Our findings demonstrate the efficacy of EpCAM-directed CAR T-cell after intracerebral administration, resulting in a reduced tumor growth and prolonged survival. However, additional systemic anti-PD-1 treatment did not increase the intratumoral persistence or the anti-tumor effects of CAR T-cell.

Locoregional injection of CAR T-cell into the surrounding brain tissue not only resulted in a noteworthy reduction in tumor growth but also achieved complete regression in selected cases. Consequently, mice receiving EpCAM-directed CAR T-cell showed significantly prolonged survival rates compared to control animals. These outcomes substantiate the findings established in our prior investigations using this model and are in line with similar observations in different tumor entities, including medulloblastoma or ependymoma [16, 32].

For T-cell suppression within the brain TME, the PD-1/PD-L1 axis has been show to play a pivotal role [33, 34]. Antigen contact induces CAR T-cell effector function and production of IFN-γ. Next, IFN-γ binds to its receptor initiating the JAK/STAT signaling pathway, which regulates PD-L1 expression on brain tumor cells and tumor-associated macrophages [35]. Accordingly, first clinical data indicate that anti-EGFRVIII-CAR T-cell infusion can paradoxically promote immunosuppressive tumor microenvironment via upregulating inhibitory immune checkpoint molecules in glioblastoma [36]. Interestingly, a phase I clinical trial investigating repeated peripheral infusions of anti-EGFRVIII CAR T cell in combination with pembrolizumab was not effective in glioblastoma [37].

Several publications highlight the CNS penetrance and effectiveness of ICB antibodies in brain metastasis. Anti-PD-1 treatment may reverse the immunosuppression within the TME, and CNS tumors have been shown to respond to combined immune checkpoint blockade, resulting in elevated proportions of tumor-infiltrating lymphocytes (TILs) [27, 38, 39]. Within the context of CAR T-cell treatment, PD-1 suppression can be achieved through the co-administration of PD-1 targeting monoclonal antibodies or the PD-1 gene editing of CAR T-cell [40].

In systemic tumor models, the additional value of PD-1 blockade to increase CAR T-cell efficacy has been debated. In a murine preclinical model for systemic melanoma, concurrent PD-1 blockade notably increased the persistence and efficacy of CAR T-cell treatment [41]. However, in another study using a immunocompetent murine model for systemic melanoma, PD-1 blockade primarily mediates its anti-tumor effect through endogenous T-cell and did not increase the anti-tumor effect of CAR T-cell treatment [42]. Furthermore, it has been demonstrated that PD-1 silencing may impair the anti-tumor function of CAR T-cell by inhibiting proliferation activity in a murine model of systemic NSCLC [43]. Song et al. demonstrated anti-EGFRVIII CAR T-cell therapy with PD-1 checkpoint blockade in a CNS tumor model using U87 glioma cells [44]. However, it is noteworthy that these experiments were conducted in immunodeficient mice, which may overlook the influence of endogenous T-cell immunity and an intact PD-1–PD-L1 signaling axis. In our study, we used autologous splenocytes for CAR T-cell production and observe no significant difference in intratumoral densities of CAR T-cell and CD3^+^T-cell, nor in CAR T-cell persistence and survival following the co-administration of anti-PD-1 and CAR T-cell. Transduction with the retroviral CAR vector endows CAR T-cell with dual specificity via the CAR and the endogenous T-cell receptor (TCR). Although CAR T-cell-based therapies are recommended for the treatment of hematological malignancies, the effects of endogenous TCR signaling in CAR T-cell biology have not been well defined. Recent preclinical and clinical studies suggest that endogenous TCR signaling is not required for CAR T-cell effector function, whereas it could negatively affect proliferation and effector function [45, 46].

Another potential mechanism contributing to the limited efficacy of the combinatorial approach is the complex composition of the tumor microenvironment (TME) within the brain which frequently harbors fewer proliferating immune cells compared to primary tumors and other metastatic sites. Additionally, T-cell in brain metastases exhibit elevated expression levels of immune checkpoint proteins compared to those in other sites, while macrophages in the brain are more prone to expressing an immune-suppressing M2 gene signature. These factors collectively contribute to impeding the effectiveness of CAR T-cell in CNS tumors [47–49].

By utilizing repetitive in vivo TPLSM, we are capable of elucidating intratumoral CAR T-cell dynamics from early stages of tumor formation until late timepoints, when large tumors have formed. After intracerebral injection of CAR T-cell, we initially observed higher intratumoral densities of EpCAM-directed CAR T-cell compared to undirected CAR T-cell. In general, CAR T-cell recognize surface antigens independently from MHC restriction. Based on the intracranial administration, early contact of EpCAM-directed CAR T-cell with EpCAM-transduced LL/2 tumor cells may lead to receptor–antigen interaction inducing activation, proliferation, and the development of a cytotoxic phenotype [50, 51]. Interestingly, intratumoral CAR T-cell density and proliferation diminished during the observation period indicating insufficient CAR T-cell persistence within the tumor. Consequently, a decreasing amount of EpCAM-directed CAR T-cell was paralleled by tumor growth. Consistent with our data, several preclinical and clinical studies in other solid brain tumors observe decreasing CAR T-cell numbers and T-cell exhaustion even when a sufficient T-cell infiltration has been achieved [52, 53]. Immunologically, large tumor burden requires persistent CAR T-cell function upon repeated antigen stimulation in an immunosuppressive environment to eventually achieve tumor eradication. However, chronic antigenic stimulation by the tumor results in endogenous T-cell exhaustion characterized by loss of lytic function and cytokine secretion with simultaneous expression of inhibitory receptors like PD-1/PD-L1 [54, 55]. Consequently, we sought to elucidate the impact of concomitant anti-PD-1 treatment on CAR T-cell migration and effector function. Surprisingly, we do not observe any differences in CAR T-cell migration to and persistence within the tumor after anti-PD-1 treatment. In line with that, no survival differences could be observed between animals receiving ICB and the isotype control antibodies, respectively. In general, anti-PD-1 antibodies mainly function by disrupting the interaction between PD-1 on T-cell and PD-L1 on tumor cells. Paucity of PD-L1 on tumor cells is a well-defined factor associated with resistance to anti-PD-1 antibody treatment, while high expression usually indicates better response rates [56–58]. However, the PD-L1 expression varies among patients and between different tumor entities [59]. Furthermore, the upregulation of alternative immune checkpoints or the activation of alternative signaling pathways within tumor cells may contribute to resistance.

For instance, tumor cells may exploit pathways other than the PD-1-PD-L1 axis to evade immune surveillance. We therefore examined TIM-3 and LAG-3 expression in our model. While LAG-3 was not detected, we observed significant TIM-3 expression on the cell surface. Although TIM-3 expression levels did not exceed those of PD-1, alternative binding partners may contribute to CAR T-cell exhaustion. Supporting this, Aslan et al. hypothesized that when PD-1 is blocked by anti-PD-1 treatment, T cell suppression might be mediated by an alternative binding partner of PD-L1 on T cell [27]. PD-L1 expression remained high on the cell surface in our model, as confirmed by immunofluorescence analysis at the end of the experiment. Additionally, it has been shown that the TME favors PD-L1 expression on tumor cells in vivo compared to tumor cells grown in culture, making it unlikely that the lack or loss of PD-L1 accounts for the observed lack of response to anti-PD-1 treatment [60]. Interestingly, CD80 has been proposed as an alternative binding partner of PD-L1 on T cell, potentially suppressing T cell proliferation and activation [61].

As both PD-1 and PD-L1 are present within the TME, this pathway could be leveraged by an innovative approach: Liu et al. engineered CAR T-cell by modifying PD-1, incorporating the extracellular and transmembrane domains of PD-1 with the intracellular signaling domain of CD28. This adaptation facilitated the transformation of inhibitory signals within the TME into activating signals [62]. The resultant “switch-receptor” CAR T-cell exhibited enhanced efficacy in tumor control compared to the concurrent administration of anti-PD-1 with CAR T-cell. The conversion of multiple inhibitory signals within the TME to stimulatory signals holds significant potential for improving anti-tumor cytotoxicity. This is particularly noteworthy given the elevated expression of checkpoints, including PD-1, LAG-3, TIM-3, and TIGIT, along with their ligands, in solid brain tumors [63]. Converting the ubiquitous inhibitory signals into stimulatory signals can thereby greatly improve CAR T-cell infiltration and persistence and has to be investigated in further studies.

Although CAR T-cell therapy shows promising results in B cell malignancies, CNS affection is a common exclusion criterion in clinical trials mainly driven by fear of neurotoxicity. Additionally, most CARs targeting solid tumors use antigens shared by normal tissues, carrying the risk of on-target off-tumor toxicity. The additional use of ICB theoretically increases efficacy while also increasing the risk of toxicity. Such side effects most frequently comprise neurological symptoms, epileptic seizures, systemic immune reactions like the cytokine release syndrome (CRS), immune effector cell-associated neurotoxicity syndrome (ICANS), organ dysfunction, and death [64, 65]. Among others, the CAR construct in our model is constituted by an scFv capable of recognizing murine EpCAM in most epithelial tissues. As a pan-epithelial marker, EpCAM is homogenously expressed on the surface of healthy alveolar tissue [66]. Due to shared expression on the surface of tumor cells and healthy tissue, the risk of on-target-off-tumor reactions is significantly increased in the context auf EpCAM-directed CAR T-cell and anti-PD-1 treatment [67]. Notably, we did not observe any clinically relevant side effects in our fully immunocompetent mouse model. Nevertheless, it remains to be mentioned that especially due to the small sample size and the translational nature of our experimental setup we cannot fully predict on on-target/off-tumor reactions of our combinatorial approach.

In conclusion, we demonstrated that locally injected CAR T-cell adjacent to the tumor lead to intratumoral accumulation and reduced tumor growth translating into a survival benefit of ^EpCAM/GFP^CAR T-cell-treated mice. Even though additional anti-PD-1 treatment was safe and well tolerated, it does not elicit unconstrained proliferation or intratumoral persistence.

## Supplementary Information

Below is the link to the electronic supplementary material.

Supplementary Figure (A,B) Kaplan-Meier survival estimates for mice-bearing brain tumors (injected seven days prior to local (CAR) T-cell administration), subsequent to treatment with either EpCAM/GFPCAR T-cell + aPD-1 (dark green; n=8) and GFPCAR T-cell + aPD-1 (dark gray; n=8), respectively (A) GFPT-cell + IgG isotype antibody (light gray, n=8) and GFPCAR T-cell + aPD-1 (dark gray; n=8), respectively (B). Log-rank test (**p=0.0005). (C) Intratumoral (CAR) T-cell density (cells/mm3) on d4, d7 and d10 after local injection of GFPT-cell (+ IgG isotype (light gray; n=8)) and GFPT-cell + anti-PD-1 antibodies (dark gray, n=9)), respectively, as determined by two-photon laser scanning microscopy. (D) Summarized tumor areas (mm2) of n=8 animals receiving locally injected GFPT-cell + IgG (light gray) and GFPT-cell + aPD-1 (n=9, dark gray). Mean ± SEM. (E) Immunohistochemical staining of PD-L1-positive cells (human tonsil) was performed to validate the specificity of the clone used for multiplex analysis. Scale bar 100µm. (TIFF 1498 kb)

## Data Availability

No datasets were generated or analyzed during the current study.

## References

[1] Ramalingam SS, Owonikoko TK, Khuri FR (2011) Lung cancer: new biological insights and recent therapeutic advances. CA Cancer J Clin 61:91–112. 10.3322/caac.20102 21303969 10.3322/caac.20102

[2] Barnholtz-Sloan JS, Sloan AE, Davis FG et al (2004) Incidence proportions of brain metastases in patients diagnosed (1973 to 2001) in the Metropolitan Detroit Cancer Surveillance System. J Clin Oncol 22:2865–2872. 10.1200/JCO.2004.12.149 15254054 10.1200/JCO.2004.12.149

[3] Sperduto PW, Yang TJ, Beal K et al (2017) Estimating survival in patients with lung cancer and brain metastases: an update of the graded prognostic assessment for lung cancer using molecular markers (Lung-molGPA). JAMA Oncol 3:827–831. 10.1001/jamaoncol.2016.3834 27892978 10.1001/jamaoncol.2016.3834 PMC5824323

[4] Hirsch FR, Scagliotti GV, Mulshine JL et al (2017) Lung cancer: current therapies and new targeted treatments. Lancet 389:299–311. 10.1016/S0140-6736(16)30958-8 27574741 10.1016/S0140-6736(16)30958-8

[5] Locke FL, Ghobadi A, Jacobson CA et al (2019) Long-term safety and activity of axicabtagene ciloleucel in refractory large B-cell lymphoma (ZUMA-1): a single-arm, multicentre, phase 1–2 trial. Lancet Oncol 20:31–42. 10.1016/S1470-2045(18)30864-7 30518502 10.1016/S1470-2045(18)30864-7 PMC6733402

[6] Maude SL, Laetsch TW, Buechner J et al (2018) Tisagenlecleucel in children and young adults with B-cell lymphoblastic leukemia. N Engl J Med 378:439–448. 10.1056/NEJMoa1709866 29385370 10.1056/NEJMoa1709866 PMC5996391

[7] Sterner RC, Sterner RM (2021) CAR-T cell therapy: current limitations and potential strategies. Blood Cancer J 11:1–11. 10.1038/s41408-021-00459-7 33824268 10.1038/s41408-021-00459-7 PMC8024391

[8] Wang C, Li Y, Gu L et al (2023) Gene targets of CAR-T cell therapy for glioblastoma. Cancers 15:2351. 10.3390/cancers15082351 37190280 10.3390/cancers15082351 PMC10136592

[9] Akhavan D, Alizadeh D, Wang D et al (2019) CAR T cell for brain tumors: Lessons learned and road ahead. Immunol Rev 290:60–84. 10.1111/imr.12773 31355493 10.1111/imr.12773 PMC6771592

[10] Zhong S, Cui Y, Liu Q, Chen S (2020) CAR-T cell therapy for lung cancer: a promising but challenging future. J Thorac Dis 12:4516–4521. 10.21037/jtd.2020.03.118 10.21037/jtd.2020.03.118 PMC7475572 32944366

[11] Hase T, Sato M, Yoshida K et al (2011) Pivotal role of epithelial cell adhesion molecule in the survival of lung cancer cells. Cancer Sci 102:1493–1500. 10.1111/j.1349-7006.2011.01973.x 21535318 10.1111/j.1349-7006.2011.01973.x PMC3381954

[12] Kim Y, Kim HS, Cui ZY et al (2009) Clinicopathological implications of EpCAM expression in adenocarcinoma of the lung. Anticancer Res 29:1817–1822 19443410

[13] Liu Y, Wang Y, Sun S et al (2022) Understanding the versatile roles and applications of EpCAM in cancers: from bench to bedside. Exp Hematol Oncol 11:97. 10.1186/s40164-022-00352-4 36369033 10.1186/s40164-022-00352-4 PMC9650829

[14] Cui Y, Li J, Liu X et al (2022) Dynamic expression of EpCAM in primary and metastatic lung cancer is controlled by both genetic and epigenetic mechanisms. Cancers (Basel) 14:4121. 10.3390/cancers14174121 36077658 10.3390/cancers14174121 PMC9454530

[15] Li D, Guo X, Yang K, et al (2023) EpCAM-targeting CAR-T cell immunotherapy is safe and efficacious for epithelial tumors. Sci Adv 9:eadg9721. 10.1126/sciadv.adg9721 10.1126/sciadv.adg9721 PMC10691766 38039357

[16] Xu T, Karschnia P, Cadilha BL et al (2023) In vivo dynamics and anti-tumor effects of EpCAM-directed CAR T-cell against brain metastases from lung cancer. Oncoimmunology 12:2163781. 10.1080/2162402X.2022.2163781 36687005 10.1080/2162402X.2022.2163781 PMC9851202

[17] Dunn GP, Bruce AT, Ikeda H et al (2002) Cancer immunoediting: from immunosurveillance to tumor escape. Nat Immunol 3:991–998. 10.1038/ni1102-991 12407406 10.1038/ni1102-991

[18] Martinez M, Moon EK (2019) CAR T cell for solid tumors: new strategies for finding, infiltrating, and surviving in the tumor microenvironment. Front Immunol 10:128. 10.3389/fimmu.2019.00128 30804938 10.3389/fimmu.2019.00128 PMC6370640

[19] Gandhi L, Rodríguez-Abreu D, Gadgeel S et al (2018) Pembrolizumab plus chemotherapy in metastatic non-small-cell lung cancer. N Engl J Med 378:2078–2092. 10.1056/NEJMoa1801005 29658856 10.1056/NEJMoa1801005

[20] Joyce JA, Fearon DT (2015) T cell exclusion, immune privilege, and the tumor microenvironment. Science 348:74–80. 10.1126/science.aaa6204 25838376 10.1126/science.aaa6204

[21] June CH, O’Connor RS, Kawalekar OU et al (2018) CAR T cell immunotherapy for human cancer. Science 359:1361–1365. 10.1126/science.aar6711 29567707 10.1126/science.aar6711

[22] Mulazzani M, Fräßle SP, von Mücke-Heim I et al (2019) Long-term in vivo microscopy of CAR T cell dynamics during eradication of CNS lymphoma in mice. Proc Natl Acad Sci USA 116:24275–24284. 10.1073/pnas.1903854116 31712432 10.1073/pnas.1903854116 PMC6883823

[23] Karches CH, Benmebarek M-R, Schmidbauer ML et al (2019) Bispecific antibodies enable synthetic agonistic receptor-transduced T cell for tumor immunotherapy. Clin Cancer Res 25:5890–5900. 10.1158/1078-0432.CCR-18-3927 31285373 10.1158/1078-0432.CCR-18-3927 PMC7611266

[24] Lesch S, Blumenberg V, Stoiber S et al (2021) T cell armed with C-X-C chemokine receptor type 6 enhance adoptive cell therapy for pancreatic tumours. Nat Biomed Eng 5:1246–1260. 10.1038/s41551-021-00737-6 34083764 10.1038/s41551-021-00737-6 PMC7611996

[25] Osswald M, Jung E, Sahm F et al (2015) Brain tumour cells interconnect to a functional and resistant network. Nature 528:93–98. 10.1038/nature16071 26536111 10.1038/nature16071

[26] Kienast Y, von Baumgarten L, Fuhrmann M et al (2010) Real-time imaging reveals the single steps of brain metastasis formation. Nat Med 16:116–122. 10.1038/nm.2072 20023634 10.1038/nm.2072

[27] Aslan K, Turco V, Blobner J et al (2020) Heterogeneity of response to immune checkpoint blockade in hypermutated experimental gliomas. Nat Commun 11:931. 10.1038/s41467-020-14642-0 32071302 10.1038/s41467-020-14642-0 PMC7028933

[28] Siegel RL, Miller KD, Jemal A (2019) Cancer statistics, 2019. CA Cancer J Clin 69:7–34. 10.3322/caac.21551 30620402 10.3322/caac.21551

[29] Aizer AA, Lamba N, Ahluwalia MS et al (2022) Brain metastases: a Society for Neuro-Oncology (SNO) consensus review on current management and future directions. Neuro Oncol 24:1613–1646. 10.1093/neuonc/noac118 35762249 10.1093/neuonc/noac118 PMC9527527

[30] Tsakonas G, Ekman S, Koulouris A, et al Safety and efficacy of immune checkpoint blockade in patients with advanced nonsmall cell lung cancer and brain metastasis. Int J Cancer. 10.1002/ijc.34628 10.1002/ijc.34628 37334528

[31] (2023) CAR-T cell for solid tumors. Nat Biotechnol 41:588–588. 10.1038/s41587-023-01803-x 10.1038/s41587-023-01803-x 37193841

[32] Donovan LK, Delaidelli A, Joseph SK et al (2020) Locoregional delivery of CAR T cell to the cerebrospinal fluid for treatment of metastatic medulloblastoma and ependymoma. Nat Med 26:720–731. 10.1038/s41591-020-0827-2 32341580 10.1038/s41591-020-0827-2 PMC8815773

[33] Eguren-Santamaria I, Sanmamed MF, Goldberg SB et al (2020) PD-1/PD-L1 Blockers in NSCLC Brain Metastases: Challenging Paradigms and Clinical Practice. Clin Cancer Res 26:4186–4197. 10.1158/1078-0432.CCR-20-0798 32354698 10.1158/1078-0432.CCR-20-0798

[34] Scheffel TB, Grave N, Vargas P et al (2021) Immunosuppression in gliomas via PD-1/PD-L1 axis and adenosine pathway. Front Oncol 10:617385. 10.3389/fonc.2020.617385 33659213 10.3389/fonc.2020.617385 PMC7919594

[35] Garcia-Diaz A, Shin DS, Moreno BH et al (2017) Interferon Receptor Signaling Pathways Regulating PD-L1 and PD-L2 Expression. Cell Rep 19:1189–1201. 10.1016/j.celrep.2017.04.031 28494868 10.1016/j.celrep.2017.04.031 PMC6420824

[36] O’Rourke DM, Nasrallah MP, Desai A, et al (2017) A single dose of peripherally infused EGFRvIII-directed CAR T cell mediates antigen loss and induces adaptive resistance in patients with recurrent glioblastoma. Sci Transl Med 9. 10.1126/scitranslmed.aaa0984 10.1126/scitranslmed.aaa0984 PMC5762203 28724573

[37] Bagley SJ, Binder ZA, Lamrani L et al (2024) Repeated peripheral infusions of anti-EGFRvIII CAR T cell in combination with pembrolizumab show no efficacy in glioblastoma: a phase 1 trial. Nat Cancer 5:517–531. 10.1038/s43018-023-00709-6 38216766 10.1038/s43018-023-00709-6

[38] Abid H, Watthanasuntorn K, Shah O, Gnanajothy R, Efficacy of pembrolizumab and nivolumab in crossing the blood brain barrier. Cureus 11:e4446. 10.7759/cureus.4446 10.7759/cureus.4446 PMC6559690 31245230

[39] Kamath SD, Kumthekar PU (2018) Immune checkpoint inhibitors for the treatment of central nervous system (CNS) metastatic disease. Front Oncol 8:414. 10.3389/fonc.2018.00414 30319977 10.3389/fonc.2018.00414 PMC6171475

[40] Abdoli Shadbad M, Hemmat N, Khaze Shahgoli V et al (2022) A systematic review on PD-1 blockade and PD-1 gene-editing of CAR-T cell for glioma therapy: from deciphering to personalized medicine. Front Immunol 12:788211. 10.3389/fimmu.2021.788211 35126356 10.3389/fimmu.2021.788211 PMC8807490

[41] Sailer CJ, Hong Y, Dahal A, et al (2023) PD-1Hi CAR-T cell provide superior protection against solid tumors. Front Immunol 14. 10.3389/fimmu.2023.1187850 10.3389/fimmu.2023.1187850 PMC10303811 37388744

[42] Davies JS, Karimipour F, Zhang L et al (2022) Non-synergy of PD-1 blockade with T-cell therapy in solid tumors. J Immunother Cancer 10:e004906. 10.1136/jitc-2022-004906 35793866 10.1136/jitc-2022-004906 PMC9260838

[43] Wei J, Luo C, Wang Y et al (2019) PD-1 silencing impairs the anti-tumor function of chimeric antigen receptor modified T cell by inhibiting proliferation activity. J Immunother Cancer 7:209. 10.1186/s40425-019-0685-y 31391096 10.1186/s40425-019-0685-y PMC6686487

[44] Song Y, Liu Q, Zuo T et al (2020) Combined antitumor effects of anti-EGFR variant III CAR-T cell therapy and PD-1 checkpoint blockade on glioblastoma in mouse model. Cell Immunol 352:104112. 10.1016/j.cellimm.2020.104112 32305131 10.1016/j.cellimm.2020.104112

[45] Yang Y, Kohler ME, Chien CD, et al (2017) TCR engagement negatively affects CD8 but not CD4 CAR T cell expansion and leukemic clearance. Sci Transl Med 9:eaag1209. 10.1126/scitranslmed.aag1209 10.1126/scitranslmed.aag1209 PMC6944272 29167392

[46] Torikai H, Reik A, Liu P-Q et al (2012) A foundation for universal T-cell based immunotherapy: T cell engineered to express a CD19-specific chimeric-antigen-receptor and eliminate expression of endogenous TCR. Blood 119:5697–5705. 10.1182/blood-2012-01-405365 22535661 10.1182/blood-2012-01-405365 PMC3382929

[47] Nduom EK, Weller M, Heimberger AB (2015) Immunosuppressive mechanisms in glioblastoma. Neuro-oncology 17 Suppl 7:vii9–vii14. 10.1093/neuonc/nov151 10.1093/neuonc/nov151 PMC4625890 26516226

[48] Sayour EJ, McLendon P, McLendon R et al (2015) Increased proportion of FoxP3+ regulatory T cell in tumor infiltrating lymphocytes is associated with tumor recurrence and reduced survival in patients with glioblastoma. Cancer Immunol Immunother 64:419–427. 10.1007/s00262-014-1651-7 25555571 10.1007/s00262-014-1651-7 PMC4774199

[49] Zhou D, Gong Z, Wu D et al (2023) Harnessing immunotherapy for brain metastases: insights into tumor–brain microenvironment interactions and emerging treatment modalities. J Hematol Oncol 16:121. 10.1186/s13045-023-01518-1 38104104 10.1186/s13045-023-01518-1 PMC10725587

[50] Hartmann J, Schüßler‐Lenz M, Bondanza A, Buchholz CJ (2017) Clinical development of CAR T cell—challenges and opportunities in translating innovative treatment concepts. EMBO Mol Med 9:1183–1197. 10.15252/emmm.201607485 10.15252/emmm.201607485 PMC5582407 28765140

[51] Kaech SM, Ahmed R (2001) Memory CD8+ T cell differentiation: initial antigen encounter triggers a developmental program in naïve cells. Nat Immunol 2:415–422. 10.1038/87720 11323695 10.1038/87720 PMC3760150

[52] Grosser R, Cherkassky L, Chintala N, Adusumilli PS (2019) Combination immunotherapy with CAR T cell and checkpoint blockade for the treatment of solid tumors. Cancer Cell 36:471–482. 10.1016/j.ccell.2019.09.006 31715131 10.1016/j.ccell.2019.09.006 PMC7171534

[53] Brown CE, Alizadeh D, Starr R et al (2016) Regression of glioblastoma after chimeric antigen receptor T-cell therapy. N Engl J Med 375:2561–2569. 10.1056/NEJMoa1610497 28029927 10.1056/NEJMoa1610497 PMC5390684

[54] Cherkassky L, Morello A, Villena-Vargas J et al (2016) Human CAR T cell with cell-intrinsic PD-1 checkpoint blockade resist tumor-mediated inhibition. J Clin Invest 126:3130–3144. 10.1172/JCI83092 27454297 10.1172/JCI83092 PMC4966328

[55] Long AH, Haso WM, Shern JF et al (2015) 4–1BB Costimulation ameliorates T cell exhaustion induced by tonic signaling of chimeric antigen receptors. Nat Med 21:581–590. 10.1038/nm.3838 25939063 10.1038/nm.3838 PMC4458184

[56] Pitt JM, Vétizou M, Daillère R et al (2016) Resistance mechanisms to immune-checkpoint blockade in cancer: tumor-intrinsic and -extrinsic factors. Immunity 44:1255–1269. 10.1016/j.immuni.2016.06.001 27332730 10.1016/j.immuni.2016.06.001

[57] Martin AM, Nirschl TR, Nirschl CJ et al (2015) Paucity of PD-L1 expression in prostate cancer: innate and adaptive immune resistance. Prostate Cancer Prostatic Dis 18:325–332. 10.1038/pcan.2015.39 26260996 10.1038/pcan.2015.39 PMC4641011

[58] Hutarew G (2016) PD-L1 testing, fit for routine evaluation? From a pathologist’s point of view. memo 9:201–206. 10.1007/s12254-016-0292-2 10.1007/s12254-016-0292-2 PMC5165031 28058063

[59] Xu S, Tian M, Zhang X et al (2020) Distribution of PD-L1 expression level across major tumor types. JCO 38:e15176–e15176. 10.1200/JCO.2020.38.15_suppl.e15176

[60] Li HY, McSharry M, Bullock B et al (2017) The tumor microenvironment regulates sensitivity of murine lung tumors to PD-1/PD-L1 antibody blockade. Cancer Immunol Res 5:767–777. 10.1158/2326-6066.CIR-16-0365 28819064 10.1158/2326-6066.CIR-16-0365 PMC5787226

[61] Butte MJ, Keir ME, Phamduy TB et al (2007) Programmed death-1 ligand 1 interacts specifically with the B7–1 costimulatory molecule to inhibit T cell responses. Immunity 27:111–122. 10.1016/j.immuni.2007.05.016 17629517 10.1016/j.immuni.2007.05.016 PMC2707944

[62] Liu X, Ranganathan R, Jiang S et al (2016) A chimeric switch-receptor targeting PD-1 augments the efficacy of second generation CAR T-cell in advanced solid tumors. Cancer Res 76:1578–1590. 10.1158/0008-5472.CAN-15-2524 26979791 10.1158/0008-5472.CAN-15-2524 PMC4800826

[63] Woroniecka K, Chongsathidkiet P, Rhodin K et al (2018) T-cell exhaustion signatures vary with tumor type and are severe in glioblastoma. Clin Cancer Res 24:4175–4186. 10.1158/1078-0432.CCR-17-1846 29437767 10.1158/1078-0432.CCR-17-1846 PMC6081269

[64] Sokolov E, Karschnia P, Benjamin R et al (2020) Language dysfunction-associated EEG findings in patients with CAR-T related neurotoxicity. BMJ Neurol Open 2:e000054. 10.1136/bmjno-2020-000054 33681787 10.1136/bmjno-2020-000054 PMC7871716

[65] Fajgenbaum DC, June CH (2020) Cytokine storm. N Engl J Med 383:2255–2273. 10.1056/NEJMra2026131 33264547 10.1056/NEJMra2026131 PMC7727315

[66] Hasegawa K, Sato A, Tanimura K et al (2017) Fraction of MHCII and EpCAM expression characterizes distal lung epithelial cells for alveolar type 2 cell isolation. Respir Res 18:150. 10.1186/s12931-017-0635-5 28784128 10.1186/s12931-017-0635-5 PMC5545863

[67] Qin D, Li D, Zhang B et al (2020) Potential lung attack and lethality generated by EpCAM-specific CAR-T cell in immunocompetent mouse models. OncoImmunology 9:1806009. 10.1080/2162402X.2020.1806009 32923168 10.1080/2162402X.2020.1806009 PMC7458607

