# Supplementary figures and images for: PD-1 blockade does not improve efficacy of EpCAM-directed CAR T-cell in lung cancer brain metastasis
Source: Cancer Immunol Immunother. 2024 Oct 3;73(12):255. doi: 10.1007/s00262-024-03837-9 (PMC11447167; doi:10.1007/s00262-024-03837-9)

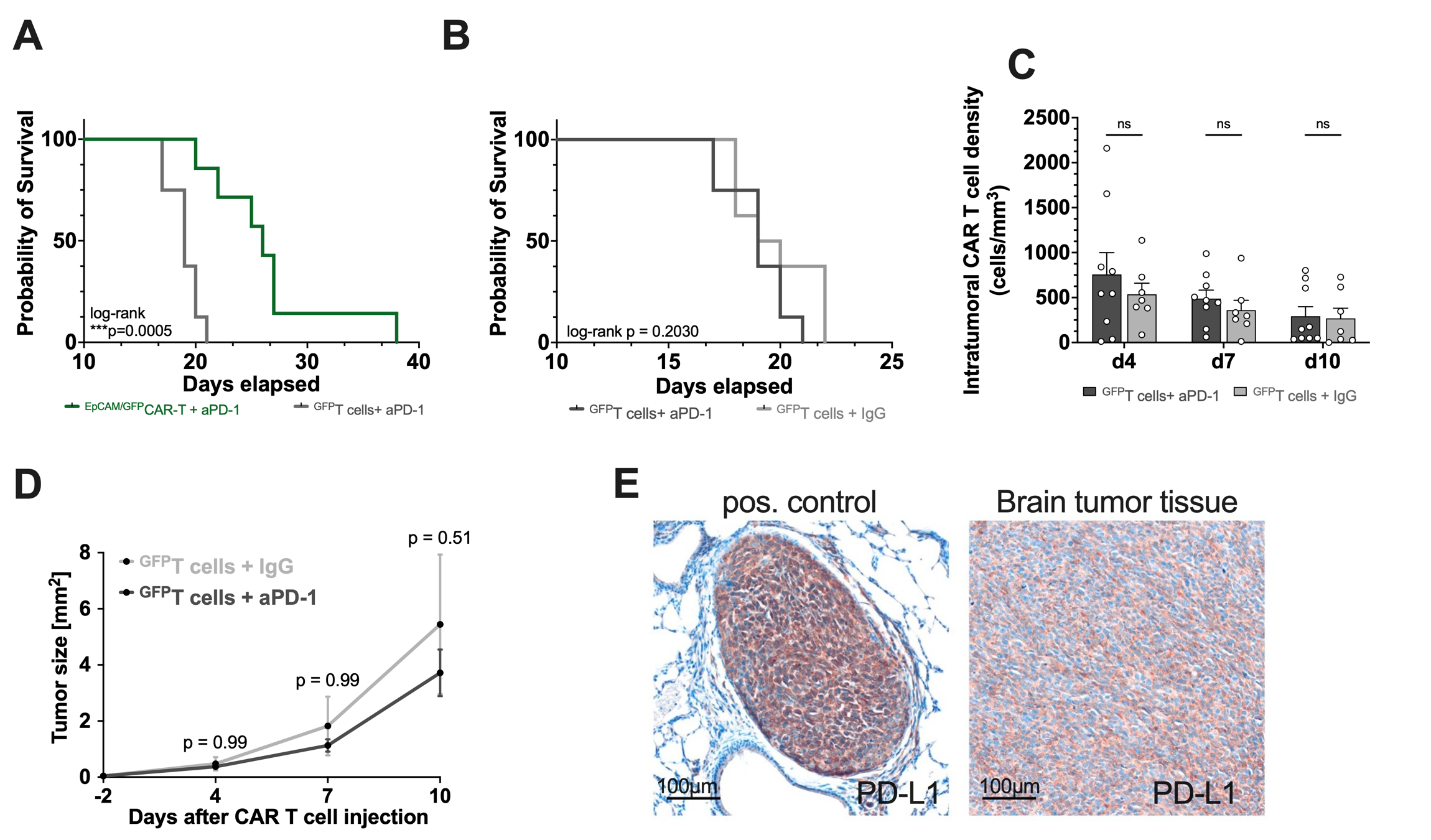

Supplement: Supplementary file 1 — Supplementary Figure (A,B) Kaplan-Meier survival estimates for mice-bearing brain tumors (injected seven days prior to local (CAR) T-cell administration), subsequent to treatment with either EpCAM/GFPCAR T-cell + aPD-1 (dark green; n=8) and GFPCAR T-cell + aPD-1 (dark gray; n=8), respectively (A) GFPT-cell + IgG isotype antibody (light gray, n=8) and GFPCAR T-cell + aPD-1 (dark gray; n=8), respectively (B). Log-rank test (**p=0.0005). (C) Intratumoral (CAR) T-cell density (cells/mm3) on d4, d7 and d10 after local injection of GFPT-cell (+ IgG isotype (light gray; n=8)) and GFPT-cell + anti-PD-1 antibodies (dark gray, n=9)), respectively, as determined by two-photon laser scanning microscopy. (D) Summarized tumor areas (mm2) of n=8 animals receiving locally injected GFPT-cell + IgG (light gray) and GFPT-cell + aPD-1 (n=9, dark gray). Mean ± SEM. (E) Immunohistochemical staining of PD-L1-positive cells (human tonsil) was performed to validate the specificity of the clone used for multiplex analysis. Scale bar 100µm. (TIFF 1498 kb) [file 262_2024_3837_MOESM1_ESM.tiff]
